# Temozolomide and cisplatin in relapsed/refractory acute leukemia

**DOI:** 10.1186/1756-8722-2-21

**Published:** 2009-05-22

**Authors:** Karen Seiter, Sreedhar Katragadda, Doris Ponce, Muhammad Rasul, Nasir Ahmed

**Affiliations:** 1 Department of Medicine, New York Medical College, Valhalla, New York 10595, USA

## Abstract

Cisplatin depletes MGMT and increases the sensitivity of leukemia cells to temozolomide. We performed a phase I study of cisplatin and temozolomide in patients with relapsed and refractory acute leukemia. Fifteen patients had AML, 3 had ALL, and 2 had biphenotypic leukemia. The median number of prior chemotherapy regimens was 3 (1–5). Treatment was well tolerated up to the maximal doses of temozolomide 200 mg/m^2^/d times 7 days and cisplatin 100 mg/m^2 ^on day 1. There was one complete remission in this heavily pretreated patient population. Five of 20 (25%) patients demonstrated a significant reduction in bone marrow blasts.

## Background

With currently available chemotherapy regimens, most patients with acute leukemia will not be cured[1,2]. There is an ongoing effort to develop new agents to treat this disease. Temozolomide is a cytotoxic alkylating agent that is approved by the United States Food and Drug Administration for the treatment of patients with newly diagnosed glioblastoma multiforme as well adult patients with refractory anaplastic astrocytoma. Preclinical studies demonstrated that temozolomide is active against a broad range of tumor cell lines, including L1210 and P388 leukemia[3,4].

Based on in vitro sensitivity of leukemia cell lines to temozolomide as well as the favorable toxicity profile of the drug, we conducted a phase I study of temozolomide in patients with relapsed and refractory acute leukemia[5]. Dose escalation occurred by increasing the number of days that patients received a temozolomide dose of 200 mg/m^2^. The dose-limiting toxicity was myelosuppression, manifested as prolonged aplasia in patients receiving 9 days of temozolomide. Extra-medullary toxicity was mild, consisting of nausea, vomiting, headache, dizziness and constipation. The recommended phase II dose of temozolomide was 200 mg/m^2^/d for 7 days. Significant anti-leukemic activity was seen in this heavily-pretreated patient population. Two patients obtained formal complete remissions (CR), and 2 other patients had complete remission without platelet recovery (CRp). In addition, 5 other patients had significant decreases in bone marrow blasts despite not obtaining a formal response (total of 9 of 20 patients had a significant decrease in bone marrow blasts).

One obstacle to temozolomide cytotoxicity is the DNA repair enzyme, O^6^-methylguanine-DNA methyltransferase (MGMT)[6,7]. Temozolomide acts predominantly through methylation of O^6^-guanine in DNA[8,9]. This results in mispairing of guanine with thymine, and, in the presence of active mismatch repair, DNA strand breaks and apoptosis[10,11]. MGMT removes these methyl groups which would have otherwise led to apoptotic cell death. Since MGMT becomes irreversibly inactivated in this process, the degree to which a cell can repair itself is inversely proportional to the level of MGMT present[12].

Laboratory studies have shown that only 25% of leukemia cells demonstrate low levels of MGMT[13]. Therefore, strategies to deplete MGMT in these cells could potentially render them more sensitive to temozolomide. One strategy is to combine temozolomide with other agents that deplete MGMT, such as cisplatin. Piccioni demonstrated that cisplatin and temozolomide were synergistic in leukemia cell lines, and that *in vivo *treatment of leukemic patients with cisplatin was followed by a reduction of MGMT activity in peripheral blood mononuclear cells[14]. D'Atri et al reported that, in Jurkat cells, cisplatin decreased MGMT activity in a time- and dose- dependent manner with maximal suppression observed 24 hours after treatment with cisplatin[15]. Thus, cisplatin is potentially one agent that could increase the efficacy of temozolomide. Based on these data we performed a phase I study of cisplatin and temozolomide in patients with relapsed and refractory acute leukemia.

## Methods

Patients with acute myelogenous leukemia (AML), acute lymphoblastic leukemia (ALL) or chronic myelogenous leukemia in blastic phase (CML-BP) that had either relapsed following, or was refractory to standard chemotherapy were eligible. Additional entry criteria included age greater than 17 years, an ECOG Performance Status of 0–3, serum bilirubin less than 1.5 mg/dl, serum creatinine < 2.0 mg/dl and a creatinine clearance greater than 60 cc/min. Patients must have recovered from any toxicity from previous chemotherapy regimens. Patients must not have received chemotherapy (other than hydroxyurea) in the 4 weeks prior to entry into this study.

All patients gave written informed consent under the guidance of the New York Medical College Institutional Review Board.

Pretreatment evaluation included a complete history and physical examination, bone marrow aspiration and biopsy for histology, cytogenetics, and flow cytometry, and routine laboratory studies including CBC with differential, chemistry profile and coagulation studies.

### Treatment

Cisplatin was administered on day 1 of therapy. The dose of cisplatin was escalated from 50 mg/m^2 ^to 100 mg/m^2 ^as in Table 1. Patients received standard hydration and antiemetics during cisplatin administration. Temozolomide was administered at a dose of 200 mg/m^2^/d, orally as a single dose on an empty stomach. The first dose of temozolomide was given 4 hours after the completion of cisplatin. The initial group of patients received temozolomide for 5 days every cycle. Patients treated at higher dose levels received 7 days of temozolomide (Table 1). Patients were eligible to receive subsequent cycles of therapy unless they had evidence of progressive disease (bone marrow blasts or peripheral blood blasts greater than pretreatment). Treatment was given every 21–28 days, provided there was no persistent non-hematologic toxicity. Patients remained on treatment until there was evidence of progression of disease. Patients who had intolerable toxicity during a course of treatment could receive subsequent cycles at one dose level lower than that at which toxicity occurred.

**Table 1:** Dose Levels

Level	n	Cisplatin	Temozolomide
1	3	50 mg/m^2^	200 mg/m^2^/d times 5 days

2	4	75 mg/m^2^	200 mg/m^2^/d times 5 days

3	5	75 mg/m^2^	200 mg/m^2^/d times 7 days

4	8	100 mg/m^2^	200 mg/m^2^/d times 7 days

Patients were entered in cohorts of 3 at the different dose levels stated. Temozolomide was increased to the dose determined in our original phase I study, and cisplatin was increased to 100 mg/m^2^.

Patients were assessed for clinical signs and symptoms of toxicity at least twice a week during the first month of treatment. Stable patients without significant toxicity in course 1 were monitored at least once a week in subsequent cycles. Patients had a bone marrow aspiration and biopsy approximately 3 weeks after treatment. Patients with obvious disease progression were not required to have this procedure. Subsequently, responding patients were to have a bone marrow aspiration and biopsy monthly for 3 months and then every 3 months until disease progression. A complete response required [1] the presence of a cellular marrow with less than 5% blasts and trilineage maturation, and [2] return of peripheral blood counts: absolute neutrophil count >1000/mm^3^, hemoglobin (untransfused) >10 gm/dl, and platelet count (untransfused) >100,000/mm^3^. Patients must have demonstrated these criteria for at least 4 weeks.

## Results

### Patient Characteristics

Twenty patients were treated on 4 dose levels of cisplatin plus temozolomide (Table 1). Sixteen patients received one cycle of therapy, three patients received two cycles, and one received three cycles. The baseline characteristics are summarized in Table 2. Fifteen patients had AML, of whom 5 patients had MDS that evolved to AML and one had aplastic anemia that evolved to AML. Five of the patients with AML had primary refractory disease. Three patients had relapsed ALL. Two patients had acute biphenotypic leukemia. One of these had primary refractory disease. Patients had received a median of 3 prior intensive chemotherapy regimens for their acute leukemia (range 1–5 treatments). The median duration of first remission for those patients who were not initially refractory was 9 months (range 3–31 months) for patients with AML, and 6 months (range 3–17 months) for patients with ALL. Three patients had relapsed after stem cell transplants (1 autologous, 2 allogeneic). Of the patients with AML, two had t(8;21), twelve had intermediate risk cytogenetics and two had poor risk cytogenetics. Of the patients with ALL, two had normal cytogenetics and one was hypodiploid. Of the patients with biphenotypic leukemia, one had complex cytogenetics and the other had t(9;22).

**Table 2:** Baseline Characteristics

	N = 20
Age	52 (24–73)
Sex	10M/10F
Performance Status	2 (1–3)
Diagnosis:	
AML	15
ALL	3
Biphenotypic	2
Number of prior regimens	3 (1–5)
Prior stem cell transplant	3 (1 auto/2 allo)
Prior AHD	6 (5 MDS/1 aplastic anemia)
Cytogenetics:	
Good Risk	2 (10%)
Intermediate Risk	13 (65%)
High Risk	5 (25%)

### Toxicity

Overall treatment was well tolerated. There were no true dose limiting toxicities. Due to the need for hydration, most patients received their chemotherapy in the hospital. The median number of hospital days was 9 (range 0–39). Hematologic toxicity is difficult to assess in this patient population. All patients had significantly abnormal blood counts at the start of therapy. There was no evidence of prolonged myelosuppression. For the limited number of patients who received more than one cycle, the median time between cycles was 21 days (range 21–28). The median number of red blood cell transfusions per cycle was 4 range (0–8) and the median number of platelet transfusions was 4 (range 0–12). Only 8 patients required intravenous antibiotics. The remaining 12 patients did not develop neutropenic fever, presumably due to the use of prophylactic oral antibiotics. For all patients, the median number of days of intravenous antibiotics was 3 (range 0–33 days); for those who did require intravenous antibiotics the median number of days was 13 (range 3–33 days).

Other grade 1/2 toxicities included nausea and vomiting in seven patients and constipation in three patients. One additional patient (treated at level 4) developed grade 3 constipation. Two patients developed grade 2 orthostatic hypotension (one patient day 8, level 2, and the other day 6, level 3). Two other patients developed asymptomatic bradycardia that occurred during chemotherapy administration (days 2–7) and resolved spontaneously. Both of these patients were treated at level 4. Due to the use of cisplatin, it was anticipated that patients would develop increases in creatinine as well as hypokalemia and hypomagnesemia. Therefore patients received hydration with supplementation of potassium and magnesium supplementation preemptively, provided they did not have hyperkalemia due to tumor lysis. Despite this, four patients developed grade 1/2 elevated creatinine (one patient level 2, three patients level 3). None of the patients treated at level 4 developed an increased creatinine, indicating that patient factors other than cisplatin dose were important in predicting this toxicity. There was no grade 3/4 renal toxicity. In all of the patients the abnormalities were rapidly reversible. Two patients treated at level 4 developed significant hypomagnesemia (grade 2) and hypokalemia (one grade 3, one grade 4). These abnormalities responded rapidly to aggressive supplementation.

### Antileukemic Effect

**Figure 1:**
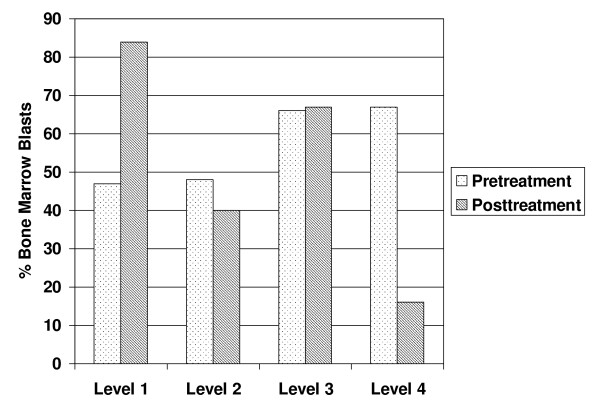
**Percentage bone marrow blasts prior to and following treatment**. The results are given for the 4 dose levels of treatment. Patients treated at level 4 had the greatest antileukemic effect.

One patient (treated at level 4) had a formal complete remission. This patient had de novo AML with normal cytogenetics. Her first remission duration (after 3 + 7 therapy) was only 3 months. She then failed to respond to idarubicin and high dose cytarabine. This patient only received one cycle of cisplatin and temozolomide; she declined further chemotherapy and expired in relapse 3 months after treatment. Two other patients (both treated at level 4) had dramatic reductions in bone marrow blasts in their bone marrow (pretreatment 69% and 87% blasts, to post-treatment 3% and 5% blasts, respectively). These patients did not meet criteria for complete remission due to a lack of peripheral count recovery. The mean percentage of blasts prior to and following treatment for the different dose levels is summarized in Figure 1. There was a trend towards increased antileukemic effect in patients treated at the highest dose level compared to the other dose levels (p = 0.07). At level 4, the mean percentage blasts in the marrow was 67% prior to treatment and 18% following treatment.

## Discussion

This study demonstrates that the combination of temozolomide and cisplatin is well tolerated in a heavily pretreated group of patients with acute leukemia. No dose limiting toxicity was seen with the addition of cisplatin to the full dose of temozolomide that was administered as a single agent in our previous study. Toxitcities were as expected and included myelosuppression, nausea, vomiting and (mild) renal and electrolyte abnormalities. We chose not to increase temozolomide beyond the dose in our other study and did not escalate cisplatin beyond that which is recommended in other malignancies.

Antileukemic activity was demonstrated, particularly at the highest dose level. Of 8 patients treated at this level, there was one complete remission and 2 other patients had 5% or fewer blasts in the bone marrow without peripheral count recovery. Although the complete remission rate at this level is only 14%, this group of patients is notoriously difficult to treat. Estey et al reported that for patients with AML in first relapse, only 11% of those whose first remission was less than 12 months achieved a complete remission with high dose cytarabine-based salvage therapy[16]. Five patients in the current study were treated as first salvage. All had a first remission duration of less than one year. Patients beyond first salvage are even more difficult to treat. Giles et al reported the outcome of 594 patients with AML undergoing second salvage therapy[17]. Overall, 13% of patients achieved a complete remission. Six adverse prognostic factors were identified: first complete remission duration less than 6 months, second complete remission duration less than 6 months, salvage therapy not including allogeneic stem cell transplant, non-inversion 16 AML, platelet count less than 50 × 10^9^/l, and leukocytosis greater than 50 × 10^9^/l. Patients were divided into prognostic groups based on the number of risk factors they had. In the current study only 3 patients were treated as second salvage. According to the Giles criteria one of them would have an anticipated CR rate of 8% and two would have an anticipated CR rate of 0%. The other AML patients treated in this study were beyond second salvage. Thus the low complete remission rate seen in our study is not unexpected.

One question is whether the addition of cisplatin to temozolomide is synergistic or at least additive. In our previous study of 20 patients who received temozolomide as a single agent there were 2 formal complete remissions (10%), and 2 complete remissions without platelet recovery (10%)[5]. In that study, nine patients (30%) had a significant decrease in bone marrow blasts. In the current study there was one complete remission (5%), and 5 patients (25%) had a significant reduction in bone marrow blasts. Only 13 patients received 7 days of temozolomide (the minimum dose in the single agent study) on the current study. In this subset, the percentage of patients with reduction in bone marrow blasts (5/13, i.e. 38%) was comparable to that seen in the single agent study. Therefore it would appear that the efficacy of the two drug regimen was comparable to the single agent regimen. However due to the small number of patients and heterogeneity of the patient groups it is impossible to draw any conclusions. A larger study would be needed to answer this question.

Another question is why cisplatin has not been used to any degree in the treatment of acute leukemia. Clearly there is in vitro data showing that some leukemia cell lines are sensitive to cisplatin [18] Complete remissions have also been reported in relapsed and refractory AML patients treated with carboplatin[19]. Undoubtedly there could be a reluctance to use an agent that causes renal and electrolyte abnormalities in a group of patients who are at high risk of these complications from their disease (tumor lysis) and supportive care (antibiotics). However with currently available supportive measures these issues are easily managed. In the current study several patients with high white blood cell counts (as high as 78,000/mm^3^) had rapid reductions in their peripheral counts. It was our impression that the reduction in peripheral counts was more rapid with the addition of cisplatin than with temozolomide alone, suggesting that the former is an active agent in this disease.

MGMT expression is an important mechanism of resistance to temozolomide. This has been demonstrated in gliomas [12] as well as in leukemia. Brandwein et al conducted a phase II study of temozolomide in poor prognosis AML patients 60 years of age or older [20]. Of 46 patients treated there were 3 complete remissions and 2 complete remissions without platelet recovery for an overall response rate of 11%. In previously untreated patients the overall response rate was 22%. Twenty-eight samples were obtained for MGMT analysis. The frequency of MGMT negativity was higher in previously untreated patients than in previously treated patients. Absent MGMT expression was significantly correlated with higher likelihood of response to temozolomide. The overall response rate was 60% for patients who were MGMT negative compared to 6% for patients with expression of MGMT. In the previously treated patients there was only one patient with no MGMT expression and that patient was the only one to attain complete remission. Caporaso et al have also added the MGMT inhibitor lomeguatrib to patients with refractory leukemia receiving temozolomide. Patients also received IL-2 subsequent to their chemotherapy. In this study six of eight heavily pretreated patients showed partial or complete disappearance of blast cells in the peripheral blood or bone marrow [21].

## Conclusion

In this phase I study in patients with relapsed/refractory acute leukemia, treatment was well tolerated up to the maximal doses of temozolomide 200 mg/m^2^/d times 7 days and cisplatin 100 mg/m^2 ^on day 1. Significant antileukemic activity was observed. Further studies with direct measurement of MGMT levels could determine which patients are likely to benefit from this therapy.

## Competing interests

KS received research support from Schering Plough for this study

## Authors' contributions

KS: Designed the study, conducted the study, collected and analyzed data and wrote the manuscript. SK: Conducted the study, collected and analyzed data, and assisted in manuscript preparation. DP: Collected and analyzed data and assisted in manuscript preparation. MR: Collected and analyzed data. NA: Collected and analyzed data
